# Supplementary material for: The Evolution and Characterization of the RNA Interference Pathways in Lophotrochozoa
Source: Genome Biol Evol. 2024 May 7;16(5):evae098. doi: 10.1093/gbe/evae098 (PMC11114477; doi:10.1093/gbe/evae098)
Supplement: evae098_Supplementary_Data [file evae098_supplementary_data.zip › Supplementary_materials.zip!/selected_miRNAs/CrGi_selected_miRNAs/CrGi_NC_047559.1_2547.pdf]

Provisional ID	:	NC_047559.1_2547
Score total	:	520.4
Score for star read(s)	:	3.9
Score for read counts	:	513
Score for mfe	:	2.4
Score for randfold	:	1.6
Score for cons. seed	:	-0.6
Total read count	:	1018
Mature read count	:	1006
Loop read count	:	0
Star read count	:	12

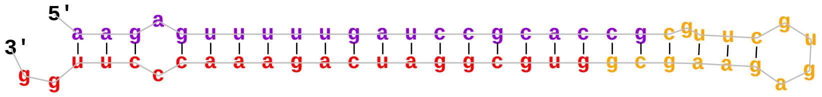

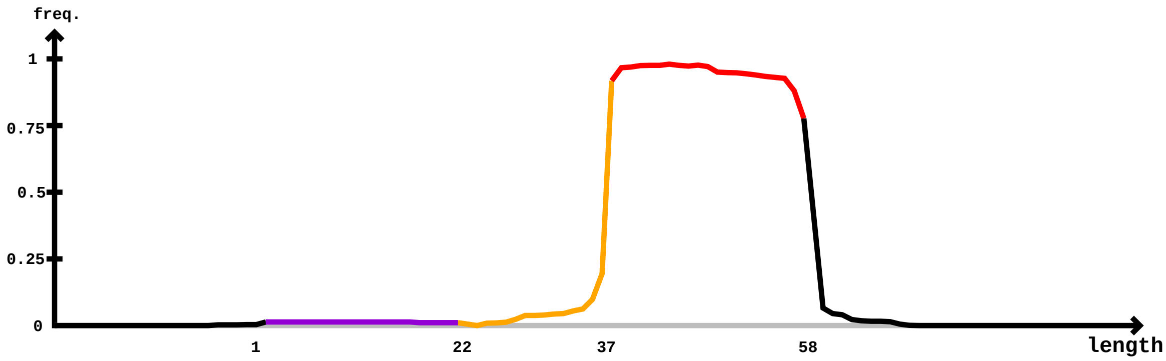

Star Mature

```
5'- agaagucaucuucguuugccaagaguuuuugauccgcaccgcguucgugagaagcggugcggaucagaaacccuuggcuagcgaagacugugaaagaaguuggaaaggaug  -3'  obs
    agaagucaucuucguuugccaagaguuuuugauccgcaccgcguucgugagaagcggugcggaucagaaacccuuggcuagcgaagacugugaaagaaguuggaaaggaug        exp
    .....(((((((((((.((((((.((((((((((((((((((.(((....))))))))))))))))))))).)))))).))))))))...)))..................     reads   mm   sample
    ....................aagaguuuuugauccgcaccg......................................................................     3       0    Ov2
    ...........................................uucgugagaagcggugcgga................................................     2       0    Ov2
    ...........................................uucgugagaagcggugcggau...............................................     2       0    Ov2
    ..............................................gugagaagcggugcggaucag............................................     3       0    Ov2
    ..............................................gugagaagcggugcggaucagU...........................................     1       1    Ov2
    ...............................................ugagGagcggugcggaucag............................................     1       1    Ov2
    ...............................................ugagaagcggugcggaucag............................................     3       0    Ov2
    ...............................................ugagaagcggugcggaucaga...........................................     1       0    Ov2
    ...................................................Aagcggugcggaucagaaac........................................     1       1    Ov2
    ..................................................gaagcggugcggaucagaaac........................................     1       0    Ov2
    ...................................................aagcggugcAgaucagaa..........................................     1       1    Ov2
    ....................................................agcggAgcggaucagaaaccc......................................     2       1    Ov2
    .....................................................gcggugcggCucagaaacccuuggcuag..............................     1       1    Ov2
    ......................................................cgUugcggaucagaaacccuu....................................     1       1    Ov2
    ......................................................cggugcggaucagaaacccuu....................................     4       0    Ov2
    ......................................................cggugcggaucagaaacccuug...................................     1       0    Ov2
    .......................................................ggugcggGucagaaacccu.....................................     1       1    Ov2
    .......................................................ggugcggGucagaaacccuu....................................     2       1    Ov2
    .......................................................ggugcggGucagaaacccuug...................................     10      1    Ov2
    .......................................................ggugcggaucagaaacccuug...................................     6       0    Ov2
    .......................................................ggugcggaucagaaacccuugg..................................     2       0    Ov2
    .......................................................ggugcggGucagaaacccuugg..................................     3       1    Ov2
    ........................................................gugcggaucagaaacccuu....................................     1       0    Ov2
    ........................................................gugcggaucagaaacccuug...................................     1       0    Ov2
    ........................................................gugcggGucagaaacccuug...................................     6       1    Ov2
    ........................................................gugcggGucagaaacccuugg..................................     107     1    Ov2
    ........................................................gugcggaucagaaacccuugg..................................     25      0    Ov2
    ........................................................gugcggaucagaGacccuugg..................................     1       1    Ov2
    ........................................................gugcggGucagaaacccuuggc.................................     12      1    Ov2
    ........................................................gugcggaucagaaacccuuggc.................................     1       0    Ov2
    ........................................................gugcggaucagaGacccuuggcu................................     1       1    Ov2
    ........................................................gugcggGucagaaacccuuggcu................................     1       1    Ov2
    .........................................................ugcggaucagaaacccuuggc.................................     7       0    Ov2
```

Star **Mature**

```
agaagucaucuucguuugccaagaguuuuugauccgcaccgcguucgugagaagcggugcggaucagaaacccuuggcuagcgaagacugugaaagaaguuggaaaggaug
.........................................................ugcggauUagaaacccuuggc.................................	1	1	Ov2
.........................................................ugcggaucagGaacccuuggc.................................	1	1	Ov2
.........................................................ugcggaucagaaacccuuggcua...............................	2	0	Ov2
.........................................................ugcggaucagaaacccuuggcuag..............................	3	0	Ov2
.........................................................ugcggaucagaaacccuuggcuagcgaag.........................	1	0	Ov2
..........................................................gcggaCcagaaacccuuggc.................................	1	1	Ov2
..........................................................gcggaucagaaacccuuggcuagcg............................	1	0	Ov2
...........................................................cggaucagaaacccuuggc.................................	1	0	Ov2
...........................................................cggaucagaaGcccuuggcua...............................	1	1	Ov2
...........................................................cggGucagaaacccuuggcuagcgaaga........................	1	1	Ov2
............................................................ggaucagaaacccuuggcuag..............................	1	0	Ov2

...............uugccaagaguuuuugauccg...........................................................................	1	0	Ov1
....................aagaguuuuugauccgcaccg......................................................................	1	0	Ov1
...........................................uucgugagaagcggugcgga................................................	1	0	Ov1
...........................................uucgugagaagcggugcggau...............................................	1	0	Ov1
..............................................gugagaagcggugcggauca.............................................	1	0	Ov1
..............................................gugagaagcggugcggaucag............................................	2	0	Ov1
..............................................gugagaagcgAugcggaucag............................................	1	1	Ov1
...............................................ugagaagcggugcggauca.............................................	1	0	Ov1
...............................................ugagaagcggugcgAaucag............................................	1	1	Ov1
...............................................ugagaagcggugcggaucag............................................	2	0	Ov1
..................................................gaagUggugcggaucagaaa.........................................	1	1	Ov1
..................................................gaagcggugcggaucagaaac........................................	1	0	Ov1
...................................................aagcggugcggaucagaaac........................................	1	0	Ov1
....................................................agcggAgcggaucagaaa.........................................	1	1	Ov1
....................................................agcggugcggaucagaaacc.......................................	1	0	Ov1
....................................................agcggAgcggaucagaaacc.......................................	1	1	Ov1
....................................................agcggAgcggaucagaaaccc......................................	2	1	Ov1
.....................................................gcggugcggaucagaaacccuug...................................	1	0	Ov1
.....................................................gcggugcggaucagaaacccuuggcuag..............................	1	0	Ov1
......................................................cAgugcggaucagaaacccuu....................................	1	1	Ov1
......................................................cggugcggaucagaaacccuu....................................	4	0	Ov1
......................................................cgUugcggaucagaaacccuu....................................	1	1	Ov1
......................................................cggAgcggaucagaaacccuu....................................	1	1	Ov1
......................................................cggugcggaucagaaacccuuggcuag..............................	2	0	Ov1
.......................................................ggugcggGucagaaacccu.....................................	1	1	Ov1
.......................................................ggugcggGucagaaacccuu....................................	2	1	Ov1
.......................................................ggugcggGucagaaacccuug...................................	3	1	Ov1
.......................................................ggugcggaucagaaacccuug...................................	3	0	Ov1
.......................................................ggugcggaucagaaacccuugg..................................	3	0	Ov1
.......................................................gguAcggaucagaaacccuugg..................................	1	1	Ov1
.......................................................ggugcggGucagaaacccuugg..................................	8	1	Ov1
........................................................gugcggaucagaaacccuu....................................	3	0	Ov1
........................................................gugcggGucagaaacccuug...................................	10	1	Ov1
........................................................gugcggaucagaaacccuugg..................................	21	0	Ov1
........................................................gugcggaucagaGacccuugg..................................	3	1	Ov1
........................................................gugcggaucagaaGcccuugg..................................	1	1	Ov1
........................................................gugcggGucagaaacccuugg..................................	85	1	Ov1
........................................................gugcggGucagaaacccuuggc.................................	10	1	Ov1
........................................................Augcggaucagaaacccuuggc.................................	1	1	Ov1
........................................................gugcggaucagaaacccuuggc.................................	1	0	Ov1
........................................................gugcggGucagaaacccuuggcu................................	2	1	Ov1
........................................................gugcggaucagaaacccuuggcu................................	1	0	Ov1
.........................................................ugcggauAagaaacccuugg..................................	1	1	Ov1
.........................................................ugcggaucagaaGcccuugg..................................	1	1	Ov1
.........................................................ugcggaucagaaacccuuggc.................................	5	0	Ov1
.........................................................ugcggaucagaaacccuuggcu................................	1	0	Ov1
.........................................................ugcggaucagaaacccuuggcuag..............................	6	0	Ov1
.........................................................ugcggaucagaaacccuuggcuagcgaag.........................	5	0	Ov1
.........................................................ugUggaucagaaacccuuggcuagcgaag.........................	1	1	Ov1
.........................................................ugcggaucagaaacccuuggcuagcgGag.........................	1	1	Ov1
.........................................................ugcggaucUgaaacccuuggcuagcgaaga........................	1	1	Ov1
.........................................................ugcggaucagaaacccuuggcuagcgaaga........................	1	0	Ov1
..........................................................Acggaucagaaacccuuggc.................................	1	1	Ov1
...........................................................cggaucagaaacccuuggcuag..............................	1	0	Ov1
..............................................................aucagaaacccuuggcuagc.............................	1	0	Ov1
..............................................................aucagaaaccAuggcuagcgaa...........................	1	1	Ov1
.................................................................agaaaccAuggcuagcgaag..........................	1	1	Ov1

...............uugccaagaguuuuugauccg...........................................................................	2	0	Mt1
```

Star **Mature**

```
agaagucaucuucguuugccaagaguuuuugauccgcaccgcguucgugagaagcggugcggaucagaaacccuuggcuagcgaagacugugaaagaaguuggaaaggaug
..................ccaagGguuuuugauccgcaccgc.....................................................................	1	1	Mt1
....................aagaguuuuugauccgcaccg......................................................................	3	0	Mt1
...........................................uucgugagaagcggugcggau...............................................	1	0	Mt1
............................................ucgugagaGgcggugcgg.................................................	1	1	Mt1
.............................................cgugagaagcggugcggauca.............................................	2	0	Mt1
..............................................gugagaagcggugcggauc..............................................	1	0	Mt1
..............................................gugagaagcggugcggaucag............................................	1	0	Mt1
...............................................ugagaagcgAugcggaucag............................................	1	1	Mt1
...............................................ugagaagcggugcggaucag............................................	4	0	Mt1
....................................................agcggAgcggaucagaaacc.......................................	2	1	Mt1
.....................................................gcggAgcggaucagaaacccuu....................................	1	1	Mt1
.....................................................gcggugcggaucagaaacccuugg..................................	1	0	Mt1
......................................................cggugcggGucagaaacccuu....................................	1	1	Mt1
......................................................cggugcggaucagaaacccuu....................................	7	0	Mt1
......................................................cggAgcggaucagaaacccuu....................................	1	1	Mt1
......................................................cggugcggaucagaaacccuug...................................	1	0	Mt1
.......................................................ggugcggGucagaaacccu.....................................	1	1	Mt1
.......................................................ggugcggaucagaaacccuu....................................	1	0	Mt1
.......................................................ggugcggGucagaaacccuu....................................	1	1	Mt1
.......................................................ggugcggaucagaaacccuug...................................	7	0	Mt1
.......................................................ggugcggGucagaaacccuug...................................	8	1	Mt1
.......................................................Agugcggaucagaaacccuug...................................	2	1	Mt1
.......................................................ggugcggaucagaaacccuugg..................................	2	0	Mt1
.......................................................ggugcggaucagaaGcccuugg..................................	1	1	Mt1
.......................................................ggugcggGucagaaacccuugg..................................	3	1	Mt1
........................................................gugcggaucagaaacccuu....................................	3	0	Mt1
........................................................gugcggGucagaaacccuu....................................	1	1	Mt1
........................................................gugcggaucagaaacccuug...................................	3	0	Mt1
........................................................gugcggGucagaaacccuug...................................	15	1	Mt1
........................................................gugcUgaucagaaacccuugg..................................	1	1	Mt1
........................................................gugcggGucagaaacccuugg..................................	171	1	Mt1
........................................................gugcggaucagaaGcccuugg..................................	1	1	Mt1
........................................................gugcggaucagaaacccuugg..................................	26	0	Mt1
........................................................gugcggGucagaaacccuuggc.................................	17	1	Mt1
........................................................gugcggaucagaaacccuuggc.................................	1	0	Mt1
........................................................gugcggGucagaaacccuuggcu................................	7	1	Mt1
.........................................................ugcggaucagaaacccuuggc.................................	1	0	Mt1
.........................................................ugcggauAagaaacccuuggc.................................	2	1	Mt1
.........................................................ugcggaucagaaacccuuggcu................................	1	0	Mt1
.........................................................ugcggaucagaaacccuuggcuU...............................	1	1	Mt1
.........................................................ugcggaucagaaacccuuggcuag..............................	1	0	Mt1
.........................................................ugcggaucagaaacccuuggcuagc.............................	1	0	Mt1
...........................................................cggaucagaaacccuuggcuag..............................	2	0	Mt1
..............................................................aucagaaaccAuuggcuagc.............................	1	1	Mt1
.................................................................aAaaacccuuggcuagcgaag.........................	1	1	Mt1
.................................................................aAaaacccuuggcuagcgaaga........................	1	1	Mt1

....................aagaguuuuugauccgcaccg......................................................................	4	0	Mt2
...........................................uucgugagaagcggugcgga................................................	2	0	Mt2
...........................................uucgugagaagcggugcggauca.............................................	1	0	Mt2
.............................................cgugagaagcggugcggauca.............................................	1	0	Mt2
..............................................gugagaagcggugcggaucag............................................	2	0	Mt2
...............................................ugagaagcggugcggaucag............................................	2	0	Mt2
.................................................agaagcgUugcggaucagaaa.........................................	1	1	Mt2
.................................................agaagcgUugcggaucagaaac........................................	1	1	Mt2
....................................................agcggugcggaucUgaaa.........................................	1	1	Mt2
....................................................agcUgugcggaucagaaacccuug...................................	1	1	Mt2
.....................................................gAggugcggaucagaaacc.......................................	1	1	Mt2
.....................................................gcggAgcggaucagaaacc.......................................	1	1	Mt2
.....................................................gcggugcggaucagaaacccuugg..................................	1	0	Mt2
......................................................cggugcggaucagaaacccu.....................................	1	0	Mt2
......................................................cggAgcggaucagaaacccuu....................................	1	1	Mt2
......................................................cggugcggaucagaaacccuu....................................	7	0	Mt2
......................................................cggugcggGucagaaacccuu....................................	2	1	Mt2
......................................................cggugcggaucagaaacccuug...................................	3	0	Mt2
......................................................cggugcggGucagaaacccuug...................................	1	1	Mt2
.......................................................ggugcggaucagaaacccuu....................................	5	0	Mt2
.......................................................ggugcggaucagaaacccuug...................................	7	0	Mt2
.......................................................ggugcggGucagaaacccuug...................................	13	1	Mt2
.......................................................ggugcggaucagaaacccuugC..................................	1	1	Mt2
```

```
                    Star                                Mature
agaagucaucuucguuugccaagaguuuuugauccgcaccgcguucgugagaagcggugcggaucagaaacccuuggcuagcgaagacugugaaagaaguuggaaaggaug
.......................................................ggugcggGucagaaacccuugg..................................   5     1     Mt2
.......................................................ggugcggaucagaaacccuugg..................................   3     0     Mt2
.......................................................Agugcggaucagaaacccuugg..................................   1     1     Mt2
.......................................................ggugcggaucagaaacccuuggc.................................   1     0     Mt2
........................................................gugcggGucagaaacccuu....................................   1     1     Mt2
........................................................gugcggGucagaaacccuug...................................   10    1     Mt2
........................................................gugcggaucagaaacccuug...................................   2     0     Mt2
........................................................gugUggaucagaaacccuug...................................   1     1     Mt2
........................................................gugcggGucagaaacccuugg..................................   172   1     Mt2
........................................................gugcggaucagaGacccuugg..................................   3     1     Mt2
........................................................gugcggaucagaaGcccuugg..................................   1     1     Mt2
........................................................gugcggaucagaaacccuugg..................................   36    0     Mt2
........................................................gugcggaucagaaacccuuggc.................................   1     0     Mt2
........................................................gugcggGucagaaacccuuggc.................................   29    1     Mt2
........................................................gugcggGucagaaacccuuggcu................................   6     1     Mt2
........................................................gugGggaucagaaacccuuggcuag..............................   1     1     Mt2
.........................................................ugcggaucagaGacccuugg..................................   1     1     Mt2
.........................................................ugcggaucagaaacccuugg..................................   1     0     Mt2
.........................................................ugcggaucagaaacccuuggc.................................   2     0     Mt2
.........................................................ugcggaucagaaacccuugCcu................................   1     1     Mt2
.........................................................ugcggaucagaaacccuuggcu................................   2     0     Mt2
.........................................................ugcggaucagaaacccuuggcuU...............................   1     1     Mt2
.........................................................ugcggaucagaaacccuuggcuag..............................   1     0     Mt2
..............................................................aucagaaaccAuuggcuag..............................   1     1     Mt2
..............................................................aucagaaacccuuggcuagc.............................   1     0     Mt2
..............................................................aucagaaacccuuggcuagcg............................   1     0     Mt2
................................................................cagaaacAcuuggcuagcgaaga........................   1     1     Mt2
.................................................................aAaaacccuuggcuagcgaa..........................   1     1     Mt2
.................................................................agaaacccuuggcuagcgaagaU.......................   1     1     Mt2
```
